# BDNF Val66Met polymorphism interacts with sex to influence bimanual motor control in healthy humans

**DOI:** 10.1002/brb3.83

**Published:** 2012-09-04

**Authors:** Ruud Smolders, Mark Rijpkema, Barbara Franke, Guillén Fernández

**Affiliations:** 1 Donders Institute for Brain, Cognition and Behavior, Radboud University NijmegenNijmegen, The Netherlands; 2 Departments of Human Genetics and Psychiatry, Radboud University Nijmegen Medical CenterNijmegen, The Netherlands; 3 Department for Cognitive Neuroscience, Radboud University Nijmegen Medical CenterNijmegen, The Netherlands; 4 Department of Psychiatry, Academic Medical Center, University of AmsterdamAmsterdam, The Netherlands

**Keywords:** BDNF, bimanual, genetics, motor, Preilowski's task, rs6265, single nucleotide polymorphism, Val66Met

## Abstract

Brain-derived neurotrophic factor (BDNF) plays a critical role in brain development. A common single nucleotide polymorphism in the gene encoding BDNF (rs6265, Val66Met) affects BDNF release and has been associated with altered learning and memory performance, and with structural changes in brain morphology and corpus callosum integrity. BDNF Val66Met has more recently been shown to influence motor learning and performance. Some of the BDNF effects seem to be modulated by an individual's sex, but currently the relationship between BDNF and sex in the motor domain remains elusive. Here, we investigate the relationship between BDNF Val66Met genotype and an individual's sex in the motor system. Seventy-six healthy, previously genotyped, individuals performed a task in which the participant drew lines at different angles of varying difficulty. Subjects controlled the horizontal and vertical movement of the line on a computer screen by rotating two cylinders. We used this bimanual motor control task to measure contributions from both current motor function and the pre-existing interhemispheric connectivity. We report that BDNF genotype interacts with sex to influence the motor performance of healthy participants in this bimanual motor control task. We further report that the BDNF genotype by sex interaction was present in the more difficult trials only, which is in line with earlier findings that genetic effects may become apparent only when a system is challenged. Our results emphasize the importance of taking sex into account when investigating the role of BDNF genotype in the motor system.

## Introduction

Brain-derived neurotrophic factor (BDNF) plays an important role in the development and maintenance of neurons and neuronal connections in the central and peripheral nervous system (Cohen-Cory et al. 2010). Activity-dependent secretion of BDNF is a necessary component for long-term potentiation (LTP) and depression processes (LTD), which are regarded as key elements of neural plasticity underlying learning and memory (Minichiello 2009).

A common functional single nucleotide polymorphism (SNP) in the gene (rs6265), leading to an amino acid change in the pro-domain of BDNF at codon 66 (Val66Met), occurs in about 30% of the human population of Caucasian ancestry (Egan et al. 2003; Hariri et al. 2003; Sen et al. 2003). The substitution of Val to Met in BDNF affects the intracellular trafficking and secretion of the BDNF protein and impairs the ability of BDNF to undergo activity-dependent release, but not general secretion (Egan et al. 2003; Hariri et al. 2003; Chen et al. 2004). Most research has focused on the effects of BDNF Val66Met on memory processes and related brain structures. Here, Met carriership has been associated with smaller hippocampal volumes (Pezawas et al. 2004; Bueller et al. 2006; Frodl et al. 2007; Karnik et al. 2010), decreased hippocampal activity, and lower declarative memory performance (Egan et al. 2003; Hariri et al. 2003).

Research on the effects of BDNF in the brain has been extended into the motor system and motor learning. Using transcranial magnetic stimulation (TMS), it was shown that BDNF Met carriers do not show the expansion of motor cortex surface area that is typically observed after a motor learning episode (Kleim et al. 2006). Cheeran et al. (2009) further elaborated on this study by showing that the LTP/LTD-like motor excitability induced with various TMS protocols is modulated by BDNF genotype, with Met carriers showing less motor cortex excitability. Met carriers were also shown to be more error prone when learning new motor skills during a delayed driving task (McHughen et al. 2010). Together, these TMS and behavioral studies provide strong evidence that BDNF genotype indeed affects motor performance and motor learning.

Recent evidence suggests that the effects of BDNF genotype may be influenced by sex (Fukumoto et al. 2010; Verhagen et al. 2010). However, a potential BDNF sex interaction in the motor domain has not yet been investigated. In this study, we tested such an interaction. As BDNF Val66Met has been shown to influence both structural brain connectivity in the corpus callosum (CC) (Chiang et al. 2011) and functional connectivity as observed with resting-state fMRI (Thomason, Yoo, Glover, & Gotlib, 2009), we use a bimanual motor task to capture possible contributions from both motor and interhemispheric motor connectivity-related processes.

## Materials and Methods

### Subjects

This study is part of the Brain Imaging Genetics (BIG) project running at the Radboud University Nijmegen (Medical Centre) (Franke et al. 2010), which is a collection of participants from (neuroimaging) studies that required genetic information. We asked all participants that had already participated in one of the studies to participate in this research, and all participants were included until the sex and genotype groups were approximately equally large. In the end, this procedure resulted in 76 highly educated (bachelor student level or higher) subjects between 18 and 35 years of age (*mean* = 23.3, *standard deviation* = 3.2, 39 women, four left handed) of Caucasian origin that reported no history of psychiatric or neurological disorders, and had normal or corrected-to-normal vision. All participants gave written informed consent and the study was approved by the local ethics committee.

### Genotyping

Saliva samples were collected from all subjects using Oragene (DNA Genotek, Kanata, Canada), and DNA extracted from these samples was used for genotyping of the BDNF (rs6265, Val66Met) SNP as described by Franke et al. (2010). The experiment leader in this study was blinded for the genotype of the participants until after data analysis.

### Experimental procedure

**Figure 1:**
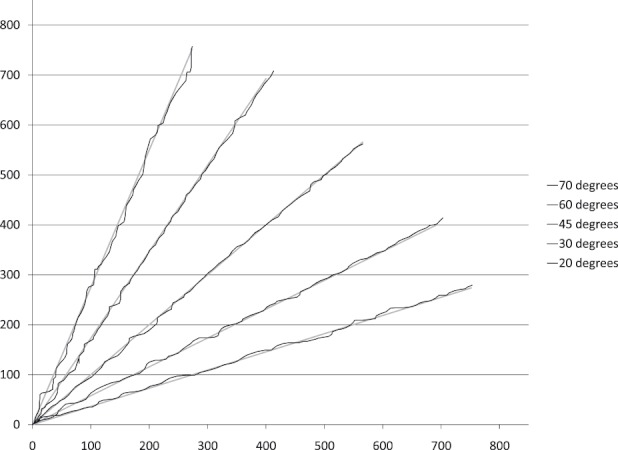
Example data of a representative subject. Data are shown for each of the angles (20°, 30°, 45°, 60°, and 70°) present in the experiment. The graph represents the computer screen with the pixels in horizontal and vertical direction indicated on the *x-* and *y*-axis. The gray lines are the example lines the subject had to mimic by simultaneously rotating two cylinders that controlled the horizontal and vertical movement.

We used a digital adaptation of Preilowski's (1972) Task, conceptually similar to the task used by Mueller et al. (2009). In this task, participants have to draw a line at a predetermined angle by simultaneously rotating two cylinders on a specially developed input device. The ability to accurately draw these lines depends on the coordination of the rotation speed of both cylinders. Participants were seated in a dimly lit room in front of a computer screen and the input device. Following instructions, the experiment consisted of 15 trials (three blocks of five trials) in which the participant had to draw a right-bound line at one of five possible angles (20°, 30°, 45°, 60°, and 70°). To indicate the predetermined angle and the length of the line the participants had to draw, a 10-pixel-wide example line was shown on the computer screen during each trial. The order of the angles was pseudorandomized, such that each angle was shown once randomly in a block of five consecutive trials, and the same angle never appeared twice in a row. The order of the angles was the same for each participant. In order to make the task more challenging for healthy participants (the original Preilowski's task was designed for patients), we included a strict time limit of 25 sec in which the 800-pixel line had to be completed, after which a 5-sec break followed. Subjects were instructed to finish drawing in time (see Fig. 1 for example data).

### Data processing

To exclude effects caused by the initial learning of the task and to keep the number of trials with a particular angle equal, we removed the first five trials of the experiment from the analysis. We removed any line-drawing data located outside of the endpoints of the example line. Subsequently, the area under the curve (AUC) score for each line was calculated by summation of the differences between the example line and the subject-drawn line for each point of the example line using custom MATLAB scripts (MATLAB 2009a; The Mathworks Inc., USA, Seattle, WA). The AUC score for each line reflects the average line-drawing error the subject made for that line. Because it has been shown that the 45° angle requires less bimanual motor control compared with the other angles (Mueller et al. 2009), we used the 45° lines as a baseline measure of performance. Because of the symmetry of the rotation movements necessary for the 60° and 30°, and 70° and 20° angles, we combined the AUC scores for both instances of these angles into two AUC scores, one for easier (60 and 30) and one for more difficult angles (70 and 20), and divided these scores by the baseline AUC score. This resulted in two baseline-corrected measures for each subject, one measure for accuracy on trials of the easier (60° and 30° angles) and one measure for the more difficult angles (70° and 20° angles). The AUC scores for easier and more difficult angles reflect the ratio between the AUC for the angles and the baseline AUC. The ratio AUC scores thus reflect how subject's performance changes due to increased task demands. The resulting AUC scores were analyzed using SPSS 16.0 (SPSS Inc., Chicago, IL).

In this experiment, we used the baseline-corrected performance on the easier and more difficult angles as within-subject variables, with BDNF genotype and sex as between-subject factors. This resulted in 2 × 2 × 2 mixed within-subject design computed using Repeated Measures ANOVA. We used Huyhn-Feldt correction when appropriate. The between-subject factors together resulted in four experimental cells, men and women homozygous for the BDNF Val-allele and men and women Met carriers. For post hoc testing, a split-file procedure from SPSS was used, which organized the output according to sex.

Data quality was ensured by applying the following procedure. Participants who failed to pass an average completion of 90% of all the lines were rejected. In contrast to the participants who had finished the lines in time, these participants may have focused more on accuracy and this could have biased our results. In order to remove outliers, AUC scores more than four times the standard deviation away from the mean of that trial over all subjects were rejected as unreliable data; this resulted in the grand total loss of five trials. Visual inspection of the resulting data showed that all trials suffering from these outlier artifacts were successfully removed. Subsequently, trials in each of the experimental cells whose scores differed by more than 2.5 times the standard deviation from the mean for that trial within that genotype group were removed from the analysis.

## Results

Of the 76 individuals entering the experiment, four subjects who had completed less than 90% of the lines and three other subjects with too many outlier data had to be excluded from the analysis. In the resulting sample of 69 participants (age 18–35; 34 women): 17 men were homozygous for the Val-allele, 16 were Val-homozygous females, and there were 18 Met-carrier men and women.

The BDNF genotype-by-sex-by-angle interaction (baseline, easier, more difficult angles) in the mixed within and between subjects 3 × 2 × 2 repeated measures ANOVA was significant (*F* (1, 65) = 4.01, *P* = 0.028). We did not observe significant main effects for sex (*F* (1, 65) = 0.74, *P* = ns) or for BDNF genotype (*F* (1, 65) = 1.8, *P* = 0.17). The between-groups BDNF by sex interaction across all angles was also significant (*F* (1, 65) = 3.95, *P* = 0.049). Because of the role of BDNF in brain maturation, we controlled for age by using age as a covariate, this covariate, however, was not significant and removing it did not change the results.

**Figure 2:**
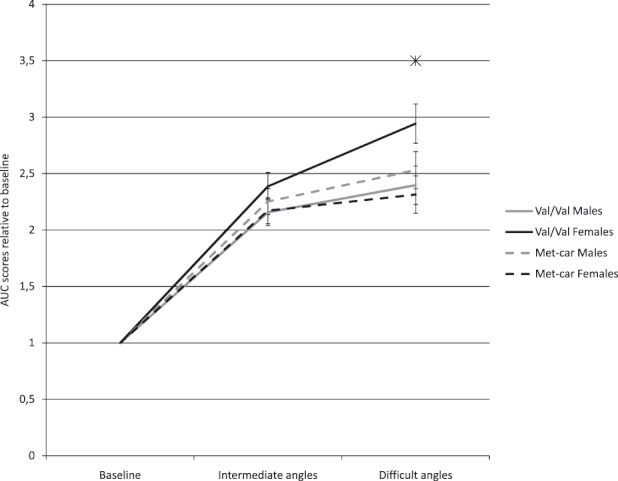
Area under the Curve (AUC) compared to baseline. The AUC relative to the baseline is shown for 45 (baseline), 60 and 30 (easier), and 70 and 20 (more difficult) degree angles. A higher score indicates less accuracy relative to baseline. For difficult angles, we show a significant interaction of BDNF genotype and sex.

To explore the BDNF genotype by sex interaction further, we performed a split-file analysis, which revealed a significant between group difference between Val-homozygous females and Met-carrier females (*P* = 0.044; see Fig. 2), especially in the most difficult angles. No such effects were observed in the male groups. These results suggest that across all angles, Val-homozygous females perform worse on the difficult angles compared with the easier angles as, expected from their baseline AUC scores.

## Discussion

We provide the first evidence that BDNF genotype and sex interact to influence the motor performance in a bimanual motor control task in females, but not in males. Interestingly, the BDNF by sex interaction was only apparent in the more difficult conditions of the task. This is striking, considering earlier work (Cousijn et al. 2010), which showed that genotype effects may only become apparent under circumstances in which the system is particularly challenged. The current findings show both the importance of taking sex into account when investigating the role of BDNF genotype, and to use challenging tasks in order to find differences that otherwise would not have been found.

Currently, most of the literature on BDNF and the motor domain consist of various measurements of motor learning, such as cortical map size (Kleim et al. 2006), motor cortex excitability (Cheeran et al. 2009), and long-term motor learning (McHughen et al. 2010). This line of research may have emerged from earlier studies on BDNF, and learning and memory processes (Egan et al. 2003; Hariri et al. 2003; Pezawas et al. 2004). The results we report here seem to contradict the existing literature for BDNF genotype effects in the motor cortex. Based on the literature, the Val66Met SNP in BDNF would be expected to selectively impair the release of BDNF during LTP/LTD-dependent learning (Minichiello 2009). We observe a difference in the normal population, which makes our finding counterintuitive. However, several reasons could help to explain our findings.

First, our study sample is considerably larger than the sample size of the other studies, making our study better able to find small differences that do not require large effect sizes, as is the case for the other studies. It could be the case that this effect has previously been missed.

Second, compared with all other BDNF motor studies performed, our task did not explicitly target motor learning, but instead it focused on immediate motor performance. Each trial took just 25 sec and the entire experiment was finished in less than 8 min, which makes LTP/LTD-based learning a less likely explanation. LTP/LTD processes need about 3 h to occur and therefore do not seem to be able to explain these immediate performance effects (Reymann & Frey, 2007). The other articles all studied BDNF genotype under motor learning conditions and used tasks that took considerably longer (Kleim et al. 2006; Cheeran et al. 2009; McHughen et al. 2010). Our counterintuitive findings thus could be explained because we tapped into a different part of the motor system, immediate performance effects caused by long-term BDNF-related changes in brain matter during development. In this line, our results do fit with the baseline difference found in the motor learning task as reported by McHughen et al. (2010).

One potential explanation for both the findings in this study and the findings of McHughen et al. (2010) could come from the idea that individual differences in bimanual motor performance are related, among others, to the structural properties of the CC. The CC is the largest interhemispheric communication pathway and plays a central role in the transfer of information from one hemisphere to the other. The integrity of the CC has been shown to be important for a variety of bimanual tasks such as Preilowski's task (Preilowski 1972), other bimanual tasks (Gerloff and Andres 2002), and simultaneous finger movements (Bonzano et al. 2008). Individual differences in CC fiber density are also associated with bimanual motor performance (Johansen-Berg et al. 2007). Recently, it was shown that there is no main effect of BDNF genotype on CC fiber density (Montag et al. 2010). However, preliminary findings we have reported previously indicate a BDNF genotype by sex interaction in the fiber density of the anterior part of the CC (Rijpkema et al., 39^th^ Annual Meeting of the Society for Neuroscience, Chicago, USA, 2009). Thus, the present results may be explained by the BDNF genotype by sex interaction that influences interhemispheric connectivity, which becomes apparent in bimanual tasks such as the one used here.

This study also fits with findings of BDNF genotype by sex interactions in other areas of research. For example, BDNF genotype effects on various aspects of behavior in female rats are dependent on the phase of the estrus cycle, confirming the notion that sex steroid hormones modulate BDNF action in females (Spencer et al. 2010). BDNF genotype by sex interactions are also found for disease vulnerability. Recently, Fukumoto et al. (2010) found that elderly female Met-carriers are more vulnerable to developing Alzheimer's disease in the later stages of life compared with males and Val-homozygous females. BDNF genotype also seems to be a risk factor for developing depression, in this case, specifically in men (Verhagen et al. 2010). While the precise mechanisms underlying these effects of BDNF on disease vulnerability are currently unknown, the role of BDNF in neuronal development and its interaction with estrogen suggest that changes in brain structure and function may be involved in both disease vulnerability and immediate motor performance.
